# Can theory of mind deficits be measured reliably in people with mild and moderate Alzheimer’s dementia?

**DOI:** 10.1186/2050-7283-1-28

**Published:** 2013-12-05

**Authors:** Caroline SM Choong, Gillian A Doody

**Affiliations:** Specialty Registrar, Mental Health Services for Older People, Nottinghamshire Healthcare NHS Trust, Cherry Ward, Highbury Hospital, Highbury Road, Nottingham, NG6 9DR England; Division of Psychiatry, Professor of General Adult Psychiatry and Medical Education, University of Nottingham, Room C22, Institute of Mental Health Building, Jubilee Campus, Triumph Road, Nottingham, NG8 1BB England

## Abstract

**Background:**

Patients suffering from Alzheimer’s dementia develop difficulties in social functioning. This has led to an interest in the study of “theory of mind” in this population. However, difficulty has arisen because the associated cognitive demands of traditional short story theory of mind assessments result in failure per se in this population, making it challenging to test pure theory of mind ability.

**Methods:**

Simplified, traditional 1st and 2nd order theory of mind short story tasks and a battery of alternative theory of mind cartoon jokes and control slapstick cartoon jokes, without memory components, were administered to 16 participants with mild-moderate Alzheimer’s dementia, and 11 age-matched healthy controls.

**Results:**

No significant differences were detected between participants with Alzheimer’s dementia and controls on the 1st or 2nd order traditional short story theory of mind tasks (p = 0.155 and p = 0.154 respectively). However, in the cartoon joke tasks there were significant differences in performance between the Alzheimer participants and the control group, this was evident for both theory of mind cartoons and the control ‘slapstick’ jokes.

**Conclusion:**

It remains very difficult to assess theory of mind as an isolated phenomenon in populations with global cognitive impairment, such as Alzheimer’s dementia, as the tasks used to assess this cognition invariably depend on other cognitive functions. Although a limitation of this study is the small sample size, the results suggest that there is no measurable specific theory of mind deficit in people with Alzheimer's dementia, and that the use of theory of mind representational models to measure social cognitive ability may not be appropriate in this population.

**Electronic supplementary material:**

The online version of this article (doi:10.1186/2050-7283-1-28) contains supplementary material, which is available to authorized users.

## Background

Theory of mind (ToM) is a relatively recent concept, first described by Premack and Woodruff (1978) and then Dennett (1978). In their study investigating the presence of theory of mind in a chimpanzee, Premack and Woodruff defined theory of mind as “being able to impute mental states to oneself and others”. In other words, it is the ability to infer other people’s mental states, thoughts and desires, and thus enables us to make predictions about behaviour. It therefore allows us to understand that people may hold different beliefs to our own and that they may act on them accordingly. Furthermore, it gives us an understanding that beliefs held by ourselves and others may not always fit with reality, i.e. we can hold false beliefs. Being able to appreciate someone else’s perspective enables us to successfully interact and communicate, and because of this theory of mind is a vital component of social cognition.

Clinical populations with impaired performance on tasks measuring theory of mind have been shown to demonstrate marked impairment in social functioning (Baron-Cohen et al. 1986). Although most research into theory of mind has been carried out with respect to autism, it has increasingly been investigated in other conditions. Various studies involving patients with brain damage to the frontal lobes have shown that patients with right frontal lesions are impaired in a variety of theory of mind tasks (Happe et al. 1999 Winner et al. 1998 Rowe et al. 2001) and bilateral damage to the orbitofrontal cortex has been associated with difficulty comprehending “faux pas” (Stone et al. 1998). There is preliminary evidence that ToM difficulties may occur in patients with Parkinsons Disease (Poletti et al. 2011), and the different forms of dementia have also been an area of research interest, particularly fronto-temporal dementia, due the markedly impaired social skills encountered in this group of patients (Gregory et al. 2002 Fernandez-Duque et al. 2009). Although perhaps not as severe as in fronto-temporal dementia, in Alzheimer’s disease, as the illness progresses, patients develop problems in social functioning and this has led to an interest into whether this may be caused by an underlying mentalising deficit.

Associations have been found between theory of mind and other aspects of cognition including verbal and performance intelligence, executive function, and information processing speed (Charlton et al. 2009). Various studies have used functional imaging and neuropsychological techniques to investigate the neural basis of theory of mind abilities in humans. They have identified a specific group of cortical regions that are reliably implicated in theory of mind, the so-called “Theory of Mind Network”. Included in these regions are the medial prefrontal cortex, the temporal poles and the temporo-parietal junction (Frith & Frith 2003 Saxe et al. 2004 Apperly et al. 2004). These brain regions are very consistent and generally identifiable in 90% of individual subjects. Alzheimer’s disease primarily affects the frontal and temporo-parietal areas of the brain, although it is associated with global atrophy of all brain regions in the latter stages.

### Assessing theory of mind

It is accepted that the gold standard test of understanding the minds of others is to grasp that they can hold false beliefs that are different from one’s own correct knowledge (Dennett 1978). Wimmer & Perner (1983) developed a paradigm that can be used with children from the age of 4 based on the case where the child’s own belief is different from someone else’s belief. The child needs to be aware that different people can have different beliefs about a situation in order to succeed on the task. The scenario they developed describes a child putting an object in a cupboard and going outside to play. While he is outside, his mother moves the object to another location. Participants are asked to predict where he will look for the object on his return.

Second order tasks test one’s ability to infer what someone else thinks about what another person thinks (Perner & Wimmer 1985). In this scenario, the same boy described above, actually witnesses his mother moving the object, but without her knowledge, and participants are asked to predict where the mother thinks her son will look for the object on his return.

Other methods that have been used include short stories involving double bluff, mistakes, persuasions or white lies (Happe 1994). More recently there have been other methods devised involving the interpretation of abstract or non-literal language, as this is a skill that is thought to require the ability to attribute appropriate mental states. For instance the interpretation of sarcasm, irony or deceit involves an understanding of what the speaker knows, believes, or intends (Baron-Cohen et al. 1999). Baron-Cohen et al. (1999) developed the faux pas test, designed for children aged 7–11, which involves the individual understanding why the speaker should not have said what he said (i.e. the faux pas), that the speaker does not realize that he has spoken in error, and why the listener would feel insulted or hurt. These tasks are felt to be more complex and subtle, however they are confounded by an increase in level of difficulty as they require understanding of non-literal language, inference of implicit meanings, recognition and understanding of complex social situations and it is difficult to control for these confounding cognitive variables. This suggests that tests such as these would not really be suitable methods for assessment of theory of mind in those populations with pre-existing cognitive impairment. Another example of alternative methods used for theory of mind assessment is the Reading the Mind in the Eyes task (Baron-Cohen et al. 1997). In this task subjects view a series of eye region photographs. They are then required to select from a given number of choices what emotion it is that they see expressed in the eyes. Again, it is thought that these less traditional methods of theory of mind assessment may be drawing on other cognitive skills aside from theory of mind and therefore results need to be interpreted cautiously.

Some studies have used cartoons to test theory of mind in other conditions, which eliminates any linguistic demand from the task. It also negates demands on working memory. This method has been applied by a number of authors in studying theory of mind, for example Corcoran et al. (1997) who used visual jokes presented as cartoon drawings in their study of mentalising ability in adults with schizophrenia as these tasks were short, enjoyable and undemanding of other skills that were compromised by the illness such as logical memory and concentration. The methodology involved presenting participants with a selection of visual jokes which were either ‘physical/slapstick’ jokes, or those which required an understanding of theory of mind to interpret. This technique has also been employed by Gallagher et al. (2000), who used cartoon jokes in a functional neuroimaging study investigating theory of mind. These same jokes were used in a number of other studies assessing theory of mind in populations with fronto-temporal dementia, Huntington’s disease and motor neurone disease (Snowden et al. 2003 Gibbons et al. 2007), as well as a further study in patients with schizophrenia (Marjoram et al. 2005).

### Theory of mind in Alzheimer’s disease

It is widely accepted that theory of mind tests are mentally demanding and that performance may be impaired by executive deficits and cognitive impairment (Saltzman et al. 2000). As an example, second order false belief tasks involve such cognitive abilities as being able to integrate relational information, retain information in working memory and filter out distracting and non-relevant information. This is obviously a major hurdle in the study of theory of mind in Alzheimer’s dementia.

Cuerva et al. (2001) compared patients with Alzheimer’s dementia to healthy controls using a short scenario assessing 1st and 2nd order false belief, as well as short stories involving various scenarios e.g. lie, joke, misunderstanding and sarcasm. They found a significant difference in performance of their subjects on the 2nd order short story task. The authors acknowledged the long and complex nature of these tasks in particular and noted that those who failed had more severe cognitive deficits than those who passed.

Gregory et al. (2002) compared patients with Alzheimer’s dementia with normal controls and patients with frontotemporal dementia across a range of tasks encompassing 1st and 2nd order false belief tasks, recognition of faux pas, and reading the mind in the eyes. The authors concluded that there was very little evidence pointing to a theory of mind deficit in Alzheimer’s dementia as the only task they performed poorly on was the 2nd order task which placed heavy memory and linguistic demands on the participants. Instead they suggested that it was more likely that the cognitively demanding nature of the 2nd order tasks is what accounted for the participants’ poor performance rather than the presence of a mentalising deficit. It should be noted however that the participants in this study were younger and higher functioning than typical patients with Alzheimer’s dementia.

Fernandez-Duque et al. (2009) followed up on this study, stating that they used the most basic ToM tasks in the literature. Again they found that the AD group performed well on 1st order tasks but were impaired when it came to the 2nd order tasks. They concluded that impaired performance in the tasks was a result of cognitive deficits rather than a mentalising ability. It was felt that the demands on e.g. reasoning, working memory and comprehension that were required by these tasks were what caused the apparent ToM impairment.

To try and elucidate the reasons for poor performance on second order tasks found in patients with Alzheimer’s dementia in previous studies, Zaitchik et al. (2004), Zaitchik et al. (2006) included control conditions in each of their several theory of mind tasks, to try to determine if the impairments were due to failure of mental state inference as opposed to inferences about information unrelated to a mental state. They also included an older and more impaired group of patients than previous studies and used the most basic tasks in theory of mind literature, which were designed initially for preschool children and which place minimal demands on language, memory and attention.

These authors found that on a range of first order tasks, Alzheimer patients were not significantly different from controls. In terms of performance on second order tasks, performance was more variable. On more traditional 2nd order false belief tasks, the Alzheimer group were significantly impaired when compared to controls, but performance was similar on both the belief questions as well as the control questions. The authors tested participants with 3 different 2nd order mental state inference tasks.

The first was a fairly traditional 2nd order false belief task in which the participant is told a short story and then asked questions that require the ability to infer what someone thinks about another person’s belief. The story was illustrated by four simple line drawings and was read aloud. Questions were asked as the story progressed to minimise demands on memory. Subjects were also given a control task that required them to make inferences not pertaining to mental states. This involved a rather complex task of making inferences regarding the location of a particular object from photographs, based on previous work done by the authors (Zaitchik 1990). These tasks were very complicated, appearing more so than the mental state inference tasks, and placed very heavy linguistic and memory demands on the participant. This is supported by the fact that the Alzheimer group did not perform as well on the control questions as they did on the mental state inference questions, even in the first order component of the task, in which they performed well. In the 2nd order component of this task they were similarly impaired across both the 2nd order mental state inference task as well as the control inference task. The authors felt that the difference between performance on the 1st and 2nd order tasks lay in demands on general cognitive processes rather than demands on processing of mental states.

In the second task the authors tested the ability of participants to make first and second order inferences about beliefs and apply them to situations involving social/moral responsibility. The authors found no significant difference between controls and those with Alzheimer’s dementia, however the results did show that both groups did worse on the 2nd order questions, which were more difficult.

In the final task the authors evaluated the participants’ understanding of the relationship between belief and behaviour – i.e. the subjects did not have to infer a character’s belief but were provided with options and were required to choose the most appropriate one. The control task involved questions relating to a photograph of a drawing depicting an object in a particular location, and the location is then changed. This again was similar to the 1st task except the participants were given options to choose from. While the Alzheimer groups did tend to perform more poorly than the controls, the only results that reached statistical significance were in the control 2nd order task, which again were attributed to the very heavy cognitive demands of the tasks.

### Study aims

As can be seen these previous studies have been hampered by the difficulty in interpreting the results of standard ToM tests due to confounding cognitive impairments. Our aim was therefore to determine if it was possible to simplify the “gold standard” false belief task even further to determine if these simplifications would enable a group of patients with AD to successfully perform the task, in particular the more complex 2nd order task. In addition, this study seeks to trial the visual joke task, which negates any demand on working memory, in the assessment of theory of mind in Alzheimer’s dementia.

This study also further seeks to clarify if there is any relationship between performance on theory of mind tasks and brain area specific cognitive domains in Alzheimer’s dementia. This is achieved by co-administration of the CAMCOG, which is the cognitive component of the Cambridge Examination for Mental Disorders of the Elderly – revised version (CAMDEX-R, Roth et al. 1998) and the Frontal Assessment Battery (FAB; Dubois and Litvan 2000), in addition to theory of mind tasks, to experimental groups.

## Methods

### Participants

The study was conducted under the auspices of the local research ethics committee. Participants with mild or moderate Alzheimer dementia (AD) and normal controls (NC) were sought from a local memory clinic. Controls were recruited from the spouses or relatives of those attending the clinic or local Alzheimer’s café, all gave written, informed consent to take part. Participants in the two AD groups either had the capacity to consent to take part, or if this was deemed lacking by the investigator (CC, a specialist in old age psychiatry), their participation was discussed with an accompanying relative, who was asked to sign a form stating that they had no objection to their relative taking part. All participants spoke English as their first language.

### Inclusion and exclusion criteria

All participants with Alzheimer’s dementia met NINCDS/ADRDA criteria for probable Alzheimer’s Dementia (McKhann et al. 1984). Medical notes of potential participants were reviewed and those with a documented medical condition that may cause dementia, or those with co-morbid psychoses, depression or other mood disorders, were excluded. Alzheimer’s participants were screened using the Hachinski Ischaemic Index (Hachinski et al. 1975), which represents a brief clinical tool helpful in the “bedside” differentiation between Alzheimer’s and Vascular dementia. Its utility has been validated by meta-analysis in pathologically verified patients with dementia. A cut-off score ≤ 4 for Alzheimer’s dementia and ≥ 7 for vascular dementia has a sensitivity of 89% and a specificity of 89% (Moroney et al. 1997). Healthy controls were screened using the Mini Mental State Examination (MMSE; Folstein et al. 1975), those scoring 27 or less were excluded. The MMSE was chosen as the mode of assessment as this was the screening test being used in the memory clinic from which participants were being recruited. In non-clinical community settings the MMSE has a negative predictive value of 98.5% and therefore appropriate for use in ruling out a dementia in healthy controls (Mitchell 2009).

Participants or their relatives were questioned as to whether they had hearing or visual impairments that would impede their performance on the administered tasks, and if so were excluded.

### Assessments

AD participants were assessed using the CAMCOG section of the revised Cambridge Examination for Mental Disorders of the Elderly (CAMDEX-R, Roth et al. 1998). The CAMDEX-R is a diagnostic assessment which provides a way to identify dementia, and to differentiate it from other common disorders and the normal processes of aging consisting of a structured clinical interview, a brief neuropsychological battery (CAMCOG) and a structured interview with a relative or other informant. The CAMCOG is devised to assess all the cognitive deficits specified in operational diagnostic criteria, i.e. memory impairment, aphasia, apraxia, agnosia and executive function. Items within a cognitive domain are graded in difficulty to permit assessment of the full range of cognitive ability. The following broad areas of cognitive function are assessed: orientation, language, memory, attention/calculation, praxis, abstract thinking and perception. This was done in order to try and determine if there was any correlation between deficits in specific cognitive domains, in particular executive function, and performance on theory of mind tasks. Participants were also assessed using the frontal assessment battery (FAB) to provide an additional assessment of executive function. The FAB is a short cognitive and behavioural 6 subtest battery for the bedside screening of global executive dysfunction. The performance on the 6 subtests of the FAB gives a composite global score, which evaluates the severity of dysexecutive syndrome and suggests a descriptive pattern of executive cognitive function in a given patient (Dubois and Litvan 2000). The FAB has been shown to be useful in the examination of executive function in Alzheimer’s dementia (Castiglioni et al. 2006). The AD group was also assessed on the MMSE to enable subdivision into two severity groups, i.e. AD-Mild: MMSE score ≥ 20 and AD-Mod: MMSE score 10–19.

All cases and controls were administered the tests in the same order, first the short story theory of mind task followed by the cartoon joke tasks.

### Short story task

This is a classic first and second order theory of mind (ToM) false belief task based on Perner and Wimmer (1985). Participants are asked questions relating to a scenario, which tests their ability to attribute false beliefs to the characters in the narrative. To minimise cognitive demands and reliance on verbal memory, and to try and ensure that participants were not failing the ToM task due to cognitive deficits, several additional measures were taken. Language was standardised to simple, short and basic sentences. The scenario was depicted with dolls to enable visualisation of the story, and was narrated by the researcher. Questions were asked as the story progressed, rather than at the end. Participants only proceeded through the whole task if sequential questions were answered correctly. If control questions were answered incorrectly, the scenario was repeated only once.

The scenario itself consisted of five simple sentences relating to two children playing with a toy train. In the scenario, child A puts the toy in a location witnessed by child B and child B then leaves the room. Child A then moves the toy but unbeknownst to them, child B witnesses this. Child B then returns to the room. The participant was asked factual questions as the story progressed to determine their understanding of the narrative. A total of five questions were asked, three assessing understanding of the scenario, one assessing 1st order theory of mind and one assessing 2nd order theory of mind (see Additional file 1).

### Cartoon joke task

Participants were shown a series of 15 single image cartoons (provided by Gallagher et al. 2000) (see Additional files 2, 3 and 4). Three sets of single image cartoon jokes were chosen. Five of these cartoons depicted either slapstick or behavioural jokes, i.e. they could be understood in physical or behavioural terms (no theory of mind component). The other ten jokes required an understanding of the characters’ mental states in order to appreciate the joke, five involved ignorance or false belief (1st order theory of mind) and five involved deception (2nd order theory of mind). The jokes were presented to each participant in the same order, starting with the slapstick, ignorance or false belief, and finally deception. Participants were informed the cartoons were intended to be humorous and were asked to explain each in these terms. Responses were recorded verbatim. A score out of zero to five was assigned to each category on completion. For a correct answer to be awarded the participants were expected to use appropriate mental state language.

### Statistical analysis

Data was analysed using SPSS for Mac version 19. A one-way ANOVA was performed to ensure participants and controls were age matched. The mean scores and standard deviations of the CAMCOG domains and FAB were calculated for the Alzheimer’s subjects. Fisher’s exact test was used to investigate differences in 1st and 2nd order theory of mind between mild and moderate Alzheimer’s sub-groups and control group. In the 1st order short story task, independent t tests were performed to compare those who passed and those who failed the task to determine any differences in scores on cognitive measures, with a Bonferroni correction applied as multiple comparisons were being made. Friedman’s test was used to determine any difference in performance on any subcategory of cartoon (ignorance/false belief, deception or slapstick) within each group (controls or Alzheimer’s groups). A Kruskal-Wallis test was then used to examine differences between controls, mild and moderate Alzheimer groups on the cartoon joke task. A post-hoc Mann–Whitney U test was performed to ascertain significant differences between combined groupings. Spearman’s rank order correlation (rho) with Bonferroni correction was performed to assess correlations between performance on the cartoon joke task and scores on the MMSE, specific domains on the CAMCOG and the FAB in subjects with Alzheimer’s disease. p values were set at 0.05 unless otherwise stated.

### Approval for Study

Ethics Approval granted by: National Research Ethics Service, Nottingham Research Ethics Committee 1, 1 Standard Court, Park Row, Nottingham, NG1 6GN. REC Ref no: 08/H0403/146.

Institutional sponsor: Research Innovation Services, University of Nottingham, University of Nottingham, King's Meadow Campus, Lenton Lane, Nottingham NG7 2NR. Ref no: RIS8133.

## Results

Twenty seven adults participated (AD-Mild, n = 7, AD-Mod, n = 9 and NC, n = 11). A one-way ANOVA found no significant difference between the three groups for mean age (p = 0.233). The age range was 65–87 years. The mean MMSE for the mild AD group was 22.3 (s.d. 1.4) and for the moderate AD group 16.3 (s.d. 2.2). There was an excess of females in the control group (8:3) but equal ratios in mild (4:3) and moderate (3:4) AD groups. As expected, the mean scores on the executive subscale of the CAMCOG and the FAB were higher in the mild AD group than the moderate AD group, 12.6 (s.d. 3.2), 10.9 (s.d. 4.5) and 7.9 (s.d. 5.2), 8.8 (s.d. 3.6) respectively. Table 1 shows the mean CAMCOG and FAB scores.

**Table 1:** **Mean scores from various domains of CAMCOG and Frontal Assessment Battery (FAB) for mild and moderate Alzheimer group**

	Mild AD group	Moderate AD group
Orientation score/10	7.4	4.8
Mean (SD)	(1.3)	(2.6)
Language score/30	23.9	20.1
Mean (SD)	(1.7)	(3.7)
Memory score/27	10.9	9.0
Mean (SD)	(3.1)	(3.4)
Attention & calculation score/9	6.3	4.7
Mean (SD)	(2.7)	(2.7)
Praxis score/12	10.1	8.8
Mean (SD)	(1.4)	(2.2)
Abstract score/8	5.7	2.0
Mean (SD)	(3.6)	(2.7)
Perception score/9	6.6	5.3
Mean (SD)	(1.0)	(2.1)
Total score/105	69.6	54.7
Mean (SD)	(6.7)	(11.7)
Executive function score	12.6	7.9
from CAMCOG/28	(3.2)	(5.2)
Mean (SD)		
Functional assessment	10.9	8.8
Battery (FAB) score/18	(4.5)	(3.6)
Mean (SD)		

### Between group comparison on the short story task 1st order false belief question

As numbers were small, the mild and moderate Alzheimer group results were combined for this analysis. Participants who failed any of the three reality questions were excluded from the analysis, as they were deemed to have not correctly understood the scenario and therefore theory of mind could not be assessed. Of the remainder there were two groups, those who passed the 1st order question and those who failed. All healthy controls (n = 11) completed the task and passed the reality questions, one failed the 1st order theory of mind question. In the Alzheimer group (n = 16), four subjects failed the reality questions, of the remaining twelve, seven passed the 1st order ToM question and five failed. Overall, no significant difference was found using Fisher’s exact test between the healthy control group and combined Alzheimer’s group.

### Between group comparison on the short story task 2nd order false belief question

The above analysis was replicated for the 2nd order ToM component of the task. Only those participants who successfully completed the 1st order task continued to the 2nd order question. Of the ten healthy controls continuing all passed the 2nd order ToM question correctly. From the seven subjects in the combined Alzheimer’s group who passed the 1st order task, two failed the 2nd order question and five passed. Again in this instance no significant difference was found between the Control and Alzheimer groups on performance on the 2nd order component of the Theory of Mind task (p = 0.154).

### Differences between Alzheimer’s subjects who passed and those who failed the 1st order theory of mind task

No significant differences were found between performance in the two groups in any cognitive domains, total CAMCOG, MMSE scores, or measures of executive functioning (p value set at 0.005 once Bonferroni correction applied for 11 comparisons). As only those who passed the 1st order ToM question proceeded to the second order question, it was not possible to carry out a similar analysis on the results of the 2nd order ToM component of the task as numbers were too small. We also looked for any difference in performance by age between those who passed and failed using an independent t test, and again, no significant difference was found (p = 0.410). Although the Alzheimer groups were combined for the analysis, there did not seem to be a specific pattern of those who passed being from one of the severity groups. Of the 7 who passed the task from the AD group, 4 were moderate in severity and 3 were mild. Of the 5 who failed the task, 3 were moderate in severity and 2 were mild.

### Between group comparison of performance on each type of cartoon joke

For each type of cartoon a significant difference was found in scores between the three different groups using the Kruskal-Wallis test (Slapstick p = 0.007, false belief/ ignorance p = 0.012 and deception p = 0.004). Considering mean ranks, the control group scored highest, followed by the mild AD group, and the moderate AD group obtained the lowest scores (For median scores see Table 2). Post-hoc Mann–Whitney U tests showed that this significant difference was between the Mild AD group & control group, and Moderate AD group and control group, for each type of cartoon (see Table 3).

**Table 2:** **Median scores with inter quartile range for performance on different categories of jokes in each group**

	Slapstick	False belief/Ignorance	Deception
	Median (Inter quartile range)	Median (Inter quartile range	Median (Inter quartile range)
Control group (n = 11)	4 (2)	4 (2)	4 (2)
Mild Alzheimer group (n = 7)	2 (2)	2 (1)	1 (1.5)
Moderate Alzheimer group (n = 9)	1 (1)	1 (2)	0 (1)

**Table 3:** **Post-hoc Man-Whitney U tests for cartoon joke task p values by groupings**

	p values	p values	p values
	Slapstick	False belief or ignorance	Deception
Mild AD vs. control	0.047	0.021	0.014
Moderate AD vs. control	0.003	0.011	0.003
Mild AD vs. moderate AD	0.249	0.344	0.315

It has been suggested in some studies, that jokes involving deception are harder to comprehend than slapstick jokes. However using the Friedman test, no significant differences were found in scores for the three different types of jokes in any of the three experimental groups (mild AD p = 0.119, moderate AD p = 0.143 and control p = 0.355).

### Correlations between scores on cartoon jokes and specific cognitive domains

Spearman’s rank order correlation (rho) to which a Bonferroni correction was then applied was used to determine if there was any correlation between performance on the cartoon joke tasks and scores on the MMSE, specific domains on the CAMCOG and measures of executive functioning. None of these reached statistical significance.

## Discussion

### Traditional short story ToM tasks and Alzheimer’s disease

Using the false belief short story task, no significant differences were found in performance on the question evaluating 1st order ToM between those with Alzheimer’s dementia and healthy age matched controls. This is in line with findings from previous studies (Gregory et al. 2002 Zaitchik et al. 2004 Zaitchik et al. 2006 and Fernandez-Duque et al. 2009). The Alzheimer subjects included in the majority of these earlier studies were less impaired and younger than those in the present study. However, Zaitchik et al. (2004) intentionally included older and more impaired subjects. Therefore the findings in our study would seem to be in keeping with those of earlier studies, i.e. there remains no demonstrable 1st order ToM deficit in Alzheimer’s dementia, as assessed by this traditional method. Furthermore, it finds no 1st order ToM deficits in individuals with differing severity of Alzheimer’s disease.

This study also found no significant differences between performance on the more complex 2nd order ToM tasks between normal controls and those with Alzheimer’s dementia. This was not replicated in other studies (Cuerva et al. 2001 Gregory et al. 2002 Zaitchik et al. 2004 Zaitchik et al. 2006 Fernandez-Duque et al. 2009). It was assumed that the tasks were too difficult for subjects to carry out, because they placed demands on cognitive domains other than theory of mind ability. One of our aims had therefore been to try and determine whether by simplifying these tasks further, those with AD would in fact be able to pass the more complex 2nd order tasks. Extra measures were taken in the administration of this task during the present study to ensure that additional cognitive demands were minimised, and this may be why no differences are apparent. This therefore adds support to the findings of these earlier trials, i.e. that it is cognitive demands other than purely mentalising ability that causes participants with Alzheimer’s dementia to fail such traditional ToM tasks. What does have to be borne in mind however is that our numbers were very small, which limits the statistical robustness of our findings and therefore further studies are needed to determine whether these results would be borne out with larger numbers included.

### Cartoon joke task and Alzheimer’s disease

For the cartoon task there were statistically significant differences between the three groups for each category of joke. However, as both AD groups were impaired on the control slapstick jokes in addition to the mental state ToM jokes, it cannot be inferred that this is secondary to a theory of mind deficit. Generally the errors made related to incorrect interpretation of the picture, or no inference being drawn as to why the cartoon was humorous.

Although the cartoon jokes eliminated the cognitive demands of working memory and the need to retain the details of a given scenario, they have brought in other cognitive demands, such as putting together the different components of an image, an appreciation of humour, and the ability to synthesize information and make an appropriate inference. Although previous authors felt this task negated any linguistic demands, there are clear demands on language abilities, as the participant is required to communicate their answer in articulate terms for it to be counted as a correct answer and be given a correct score. It may be that the Alzheimer group simply found it more difficult to articulate the mental states of the characters in the jokes, which is potentially more a function of language, rather than ToM ability. We know that discourse declines significantly in the course of AD, becoming increasingly unorganized and empty, with uninformative speech, a great number of indefinite terms, meaningless sentences and the absence of relevant elements for the comprehension of the message expressed observed, as the disease progresses (Brandao et al. 2009). What this finding does demonstrate however, is that the use of these cartoon jokes is not appropriate in the assessment of ToM in AD.

### Cognitive tests

No significant differences were found in performance on cognitive tasks between those who passed and those who failed the 1st order ToM short story task. Statistical analysis was performed to try and determine if there was any correlation between performance on the cartoon joke task and MMSE, scores on each domain measured by the CAMCOG, total CAMCOG score and Frontal Assessment Battery score. If correlated, this may have given some indication as to other cognitive areas that may be impaired in the task completion. However, none of these correlations reached statistical significance. It is accepted however, that the significance level in measures of correlation are strongly influenced by sample size and therefore for small samples there may be moderate correlations that do not reach statistical significance (Pallant 2001). Equally, large numbers of correlations between experimental and cognitive measures do also increase the potential for type 1 errors, and therefore it is difficult to draw any firm conclusions in this regard from our results. It may be that larger numbers and a more robust neuropsychological assessment may have yielded significant correlations in contrast to the CAMCOG.

## Conclusion

The sample size of this study is clearly a major limitation in terms of being able to draw very strong conclusions from our findings. While we acknowledge that further study is required to confirm these findings, we feel that our study does add to the body of evidence that patients with AD do not have a specific ToM deficit as can be demonstrated by current tests. Perhaps more significantly, we have demonstrated that the alternative method of assessing ToM using cartoon jokes is not a suitable test to use in this population. It is challenging to measure theory of mind in isolation, without drawing on other cognitive requirements. The current tests would not be suitable for use in a significant proportion of AD patients, as highlighted by the numbers that had to be excluded for failing control questions in the short story task. Furthermore, these tests have not been assessed in those with neuropsychiatric symptoms which as we know commonly occur in AD. While it is without doubt a highly interesting research question, it would seem that there is limited clinical application of these tests in day to day practice.

## Electronic supplementary material

Additional file 1: **Short story task.** (DOC 31 KB)

Additional file 2: **Example of Slapstick Cartoon.** (DOC 183 KB)

Additional file 3: **Example of False Belief/Ignorance Cartoon.** (DOC 87 KB)

Additional file 4: **Example of Deception Cartoon.** (DOC 188 KB)
