# Supplementary material for: Can theory of mind deficits be measured reliably in people with mild and moderate Alzheimer’s dementia?
Source: BMC Psychol. 2013 Dec 5;1(1):28. doi: 10.1186/2050-7283-1-28 (PMC4269983; doi:10.1186/2050-7283-1-28)
Supplement: Supplementary file 1 — Additional file 1: Short story task. (DOC 31 KB) [file 40359_2013_22_MOESM1_ESM.doc]

**Short Story Task**

Scenario read aloud to participants (depicted by bold font)

Sam and Daisy are playing with a toy train in their bedroom. They finish playing and put the train away in the toy chest. Daisy then leaves the room.

*1^st^ factual question is asked: Where is the train?*

*Answer: In the toy chest*

Sam then moves the train from the toy chest and hides it under the bed.

*2nd factual question is asked: Where is the train now?*

*Answer: Under the bed*

*1^st^ order TOM question is asked: Where will Daisy look for the train when she comes back into the room?*

*Answer: The toy chest*

In actual fact, while Tom was hiding the train, Daisy was secretly peeking back into the room and saw him hide it under the bed.

*3^rd^ factual question is asked: Does Sam know Daisy was watching him hide the train?*

*Answer: No*

*2^nd^ order TOM question is asked: Where does Sam think that Daisy will look for the train when she returns to the room?*

*Answer: In the toy chest*
