# Supplementary material for: Can theory of mind deficits be measured reliably in people with mild and moderate Alzheimer’s dementia?
Source: BMC Psychol. 2013 Dec 5;1(1):28. doi: 10.1186/2050-7283-1-28 (PMC4269983; doi:10.1186/2050-7283-1-28)
Supplement: Supplementary file 2 — Additional file 2: Example of Slapstick Cartoon. (DOC 183 KB) [file 40359_2013_22_MOESM2_ESM.doc]

Example of Slapstick Cartoon

**Example:**

**Correct response:** *The cat is using the man’s wooden leg as a scratch post.*

**Incorrect response:** *The gentleman is smoking a pipe in a rocking chair. He hasn’t got a foot on one side.*
