# Supplementary material for: Can theory of mind deficits be measured reliably in people with mild and moderate Alzheimer’s dementia?
Source: BMC Psychol. 2013 Dec 5;1(1):28. doi: 10.1186/2050-7283-1-28 (PMC4269983; doi:10.1186/2050-7283-1-28)
Supplement: Supplementary file 3 — Additional file 3: Example of False Belief/Ignorance Cartoon. (DOC 87 KB) [file 40359_2013_22_MOESM3_ESM.doc]

Example of False Belief / Ignorance Cartoon

**Example:**

**Correct response:** *The poor man is distracted by the piano stool not realizing the whole piano is going to fall on him.*

**Incorrect response:** *The person there has a piano stool beside them as though it’s collapsed. He’s looking at it puzzled. The grand piano is opened up to be played*.
