# Supplementary material for: Can theory of mind deficits be measured reliably in people with mild and moderate Alzheimer’s dementia?
Source: BMC Psychol. 2013 Dec 5;1(1):28. doi: 10.1186/2050-7283-1-28 (PMC4269983; doi:10.1186/2050-7283-1-28)
Supplement: Supplementary file 4 — Additional file 4: Example of Deception Cartoon. (DOC 188 KB) [file 40359_2013_22_MOESM4_ESM.doc]

Example of Deception Cartoon

**Example:**

**Correct response:** *The young couple are having a cuddle. The boy is hitting a ping pong ball up and down to make the mother think they are having a game of ping pong.*

**Incorrect response:** *A lady is sitting in an armchair reading. In the adjoining room there is a table tennis table. There is a man with a table tennis bat and a young lady sat on his knee.*
